# A Practical Treatise on Diseases of Children

**Published:** 1885-11

**Authors:** 


A PRACTICAL TREATISE ON THE DISEASES OF CHILDREN. By Alfred Vogel, M. D., Professor of Clinical Medicine in the University of Dorpat, Russia. Translated and edited by H. Raphael, M. D., formerly house Surgeon to Bellevue Hospital; Physician to the Eastern Dispensary for the Diseases of Children; attending Physician Bellevue Hospital, Out-Patient Department, Diseases of Genito-Urinary Organs and Syphilis, etc. Third American from the eighth German edition. Revised an enlarged. Illustrated by six lithographic plates. New York: D. Appleton & Co., 1, 3 and 5 Bond street. 1885.

These six hundred and forty pages of German lore ought to reward the reader who is desirous of knowing how babies are treated abroad, and the additions and notes of the American editor on cerebro-spinal meningitis and other matters should meet the demand in the line of Pædiatrics at home.

The elegant plates at the close enhance the value of this large and well-gotten-up volume, which does credit to the publishers.

